# Health, Identification and Pleasure: An Ethnographic Study on the Self-Management and Construction of Taijiquan Park Culture Space

**DOI:** 10.3390/ijerph18168860

**Published:** 2021-08-23

**Authors:** Xiujie Ma, Jing Xie, George Jennings

**Affiliations:** 1 Chinese Guoshu Academy, Chengdu Sport University, Chengdu 610041, China; 2 School of Wushu, Chengdu Sport University, Chengdu 610041, China; 3 School of Humanities and Arts, Shanghai Lixin University of Accounting and Finance, Shanghai 201209, China; 20200019@lixin.edu.cn; 4 Cardiff School of Sport and Health Sciences, Cardiff Metropolitan University, Cardiff CF23 6XD, UK; gbjennings@cardiffmet.ac.uk

**Keywords:** public space, self-management, Taijiquan park cultural space, identity

## Abstract

The public space of a park is one of the most important carriers of social interaction and cultural practice in urban areas. Taking an ethnography of Taijiquan in Chengdu (China) as a case study, this article explores the production of Taijiquan’s “park culture space” (PCS). Our analysis revealed that the development of PCS not only transformed “public space” in the park to a “private space” through Taijiquan practice and exchange but also transformed “material space” in the park into “social space” with identification. We found that working on the process of self-managing Taijiquan’s “park culture space” included the democratic operation mechanism of communication and consultation, the cooperative operation mechanism of mutual benefit, and the incentive operation mechanism of balancing interests. Taijiquan’s “park culture space” was the reproduction of public space that was not only bonded with Taijiquan practice but was also reconstructed and expanded by Taijiquan practitioners. Furthermore, it involved the return of Taijiquan practitioners’ historical memory and collective life experience alongside the construction of Taijiquan practitioners’ group identity and the development of self-organization.

## 1. Introduction

Urban parks are an important part of urban public space and ecosystems. They are the primary place for urban residents to perform outdoor activities, including gathering, relaxing, keeping fit, and having fun [1]. Urban parks play an important role in urban development planning and the quality of life for residents [2]. In particular, with the development of urbanization, increasing amounts of people have moved to cities and settled down. In 2018, 55 percent of the world’s population lived in cities [3]. By the end of 2019, China’s urban permanent population reached 848.43 million, accounting for 60.6 percent of the total population [4]. Increasingly, scholars have begun to pay attention to the study of public spaces in urban parks. A large number of studies have not only argued that parks play an important role in improving the urban environment [5,6,7,8] but also that they undertake an important function in the service of sports [9,10,11,12,13]. In addition, they play a positive role in maintaining human physical and mental health. Furthermore, several studies have confirmed that the accessibility and fairness of parks affect the level, motivation, and frequency of sports activities and even the physical condition of urban residents [1,14,15,16,17].

The Chinese martial and movement art of Taijiquan (Tai Chi Chuan or “Grand Ultimate Fist”) is an example of an accessible activity for people of a wide range of ages and physical abilities that is often practised in public open spaces such as parks. It is a specific form of body culture that reflects aspects of Chinese culture, Daoist philosophy, traditional medicine, and martial history through its symbolic movements, terminology, ideology, and pedagogy. Depending on the context, it is used as a recreational exercise, a physical therapy, a form of moving meditation, and a lifestyle practice. Although it has transitioned from being a military training system to a regional, clan-based martial art and even to training protocols for actors [18], Taijiquan is best known as a globalised, gentle, healthy activity that can be used to heal people from various ailments common in contemporary societies [19].

As studies have shown, Taijiquan in general can provide good health benefits for the general public, including improving balance, attention, quality of sleep; reducing heart rate and blood pressure; increasing vagus nerve activities; lowering cholesterol; and relieving chronic disease symptoms, such as muscle pain, osteoarthritis, and rheumatoid arthritis [20]. Many of these biomedical studies were developed in tightly controlled, indoor laboratory environments rather than using outdoor spaces such as parks and other green areas so typically associated with Taijiquan groups today. This aspect of ecological validity is important; since “New China” was established in 1949, in line with support from Chairman Mao, Taijiquan in the People’s Republic of China era has rapidly developed toward standardization and simplification. At the same time, the Chinese government issued a series of policies to promote and popularize Taijiquan; thus, many elderly people in China like practicing Taijiquan to maintain their health. Specifically, in 2020, Taijiquan was added to the UNESCO’s Representative List of Intangible Cultural Heritage of Humanity [21]. Nowadays, increasingly more urban people join in Taijiquan practice in public spaces (e.g., urban parks, green places, and squares). As Yang Chengfu (one of the forerunners of modern Yang style Taijiquan) stated, the practice of Taijiquan requires a quiet environment with fresh air that is open and ventilated [22]. This why most people prefer practicing Taijiquan in China’s urban parks.

Nevertheless, focused scholarly work on urban parks and Taijiquan specifically is surprisingly sparse, with only the anthropological work of Frank (which is probably the most detailed and sustained empirical research on the urban park culture of Taijiquan) raising a range of important insights into the park lives and secret spaces of Taijiquan [23,24]. However, the research object of our study was different from the focus of Frank’s research, as we are concerned with mass Taijiquan practitioners working in large groups, while Frank’s study was on “master–disciple” inheritance practitioners, mainly of the Wu family Taijiquan. Mass Taijiquan practitioners in urban parks mostly aim toward maintaining health rather than specific martial arts skills for self-defence or Daoist self-cultivation. The content of the practice mainly focuses on how to improve physical fitness and also involves some knowledge of traditional health preservation and traditional philosophy. The teaching arrangement is relatively flexible. Conversely, using the “master–disciple” inheritance, Taijiquan practitioners not only practice hand form (Tao Lu) and weapons form (Qi Xie) but also practice pushing hands (Tui Shou) and scattering (San Shou). Certain people even practice in private. As far as the first author is aware (as a practitioner of traditionalist Taijiquan himself), in China, the inherited “master–disciple” Taijiquan teaching and practice in urban parks are becoming rarer.

## 2. Taijiquan Park Culture Space (Taijiquan PCS): Concept and Theory

As Lefebvre pointed out, “space” is the product of society; it has never been empty and always contains a certain meaning [25]. From a basic interpretation, philosophy and physics have different definitions of “absolute space” and “relational space” in the broad sense. Madanipour divided “space” into a clear, physical, and real material space, namely, “absolute space”, and “relative space” perceived by human subjective observation [26].

The concept of “public space” has been used frequently in many fields, such as geography, sociology, politics, and communications. When the academic disciplines listed above use this concept, the objects they refer to are not exactly the same and their specific connotations are also quite different. Jacobs, one of the earliest scholars to discuss this concept, introduced the term “public space” into the field of urban research and pointed out that more attention should be paid to the “public space” of “urban space”, regarding it as an important method and spatial link for reshaping interpersonal communication and creating harmonious and dynamic urban life in traditional urban blocks [27]. From the perspective of human geography, Kohn believed that the ideal public space needs to meet three characteristics: public property rights, equal access, and full social interaction [28]. Colquhoun divided urban public space into “built space” and “social space”. These concepts show that urban space is not only the material existence of urban development, including the functions of vision and use, but also the subjective experience that is related to time, movement, etc. The change and development of space not only contain objective regularity but also reflect the operative mechanism of social institutions as the product of society, politics, and economy [29]. As Carr pointed out in *Public Space*, the key to building the image of public space lies in the “relevance” between public spaces and individuals, groups, and society [30].

Public space is an important field in which members of society gather, interact, and construct social relations. Amin defined public space, such as parks, streets, and squares, in cities as the symbol of collective happiness, the place where urban people meet and urban culture forms, the field for city leaders to realize their vision of political and social governance, and the important space for public political games and negotiation [31]. With rich representations in Western culture, such as the Agora (a forum in ancient Rome) and the Speaker’s Corner in Hyde Park in London, public spaces embody grassroots social groups’ identity and appeal by facilitating gathering and performing in open fora [32,33,34,35]. At the same time, strangers can meet and interact in public spaces. The encounters, interactions, and even conflicts between social members in public spaces create diverse and complex urban cultures. The encounter and collision with “the other” create new possibilities for the construction of social relations and cultural significance, although it may be short and functional [36,37].

Drawn from the views of “social public space” listed above, it was concluded that any social fact or social action itself has specific spatial attributes. A certain society (action) directly corresponds to a certain space. As a kind of social behavior, practicing Taijiquan naturally leads to “Taijiquan’s culture space”. This kind of space is substantial and physical for sports and fitness, and it is also a social space that is an expansion and personification of the original space that is driven by the social practice of the Taijiquan group. In other words, various kinds of social relations grow in the physical space occupied by Taijiquan practitioners, and this creates a kind of social capital for Taijiquan practitioners. Taijiquan park culture space (Taijiquan PCS) is a type of social and cultural space with potentially profound implications.

We define Taijiquan PCS as a relatively fixed form of social connection and interpersonal relationship structure that is formed in specific urban public park spaces through the practice of Taijiquan. It includes the physical and material park space for practice as well as the diversified and virtual “social space” that is expanded by practicing Taijiquan. In this article, we focus on the formation process, self-management system, and its influence on the identification of specific groups. Therefore, by exploring the interaction structure and identification of Taijiquan practitioners, this study focuses on the production and transformation of Taijiquan PCS and then explains its inner mechanism, namely, a system of self-management.

## 3. Research Area and Research Method

### 3.1. Location Selection for Ethnographic Study

We selected the Chengdu People’s Park in Sichuan Province as a research case study and conducted ethnographic research using in-depth descriptive and theoretical analyses.

**Figure 1:**
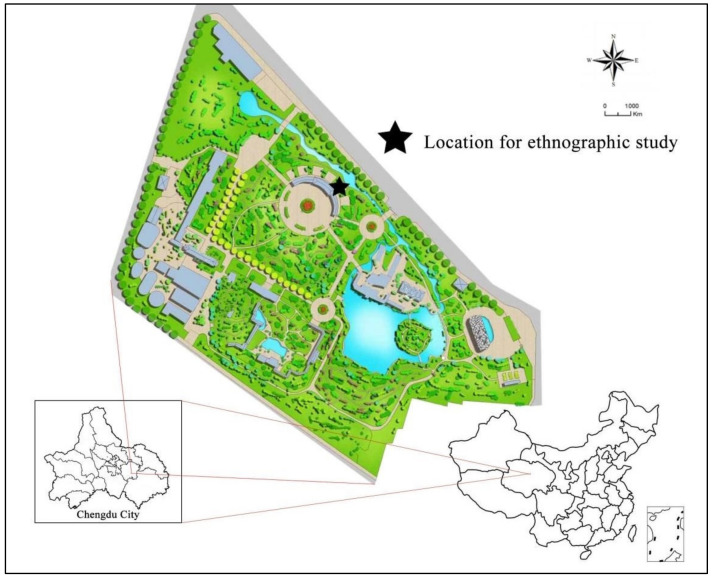
The map of Chengdu People’s Park and the location of the ethnographic study.

The People’s Park is located in Qingyang District, Chengdu, near the city center. It is approximately 700 m west of Tianfu Square and 500 m from Kuanzhai Alley, a famous tourist attraction in Chengdu. The park is surrounded by Shaocheng Road, Wenweng Road, Junping Street, and Xiaonan Street. The surrounding area is dominated by mixed residential and commercial land. The total area of the park is 131,898.42 square meters, which includes five area types: green area (52.22%), water area (9.47%), road area (25.11%), square area (7.53%), and building base area (5.66%). The People’s Park has lush trees and a quiet environment. There are four squares of different sizes, three different types of botanical display gardens, six historically protected buildings, and recreational places, such as tea houses, children’s playgrounds, and elderly people’s activity areas (see Figure 1). Chengdu People’s Park was built in 1911. It spans the three historical periods of the late Qing Dynasty, the Republic of China, and New China. It has experienced the turmoil of the times and the baptism of war. The development of Chengdu People’s Park has also witnessed the changes in Chengdu City and carries the memory of Chengdu City itself and generations of Chengdu citizens.

Due to the highly developed urbanization of Chengdu, the population around the People’s Park is highly concentrated and public space is relatively lacking. Thus, all occupants who live near the park like to come here for outdoor activities or sports. According to statistics from managers of the People’s Park, the park’s annual visitor volume is 3.2 million. The number of daily visits to the park is greatly affected by the weather. When the weather conditions are bad, the number of visitors ranges from 7000 to 8000. When weather conditions are better, it can reach up to 10,000 people. On weekends and when flower shows are held, the number of visitors can reach 20,000–30,000 [38]. The period for most regular Taijiquan practitioners who practice in Chengdu People’s Park is fixed; the training period is 6:30–8:30 in the morning and 4:30–6:30 in the afternoon.

### 3.2. Research Method

This study adopted the qualitative research method of participatory ethnography. Compared with quantitative methods, participatory ethnographic research has the characteristics of comprehensiveness, depth, and participation. Through participating in the research and collecting vivid and detailed data, it can better reflect the formation, development, and evolutionary process of Taijiquan PCS as well as its internal production and operation mechanism [39].

The first author has practiced Taijiquan for many years and has taught Taijiquan courses in one university, and he initially participated in all activities of the group as a learner and volunteer. After a period of practice and communication to obtain the practice group’s trust, the first author finally truly joined the group as a formal member and recognised insider. The survey period was from December 2020 to May 2021.

### 3.3. Data Collection

The ethnographic research method of “immersion” was adopted to participate in the daily practice and other related activities of the Taijiquan practice group. All data were collected by means of on-site notes and participant interviews. During the study period, the first author participated in all activities of the Taijiquan practice groups in Chengdu People’s Park. In addition to recording their activities and conversations, we also made appointments and conducted focus group interviews or individual ones at each site. The focus group interviews were usually conducted when the practice was finished and usually lasted 1 h. Each interview was carried out in one of two forms: a random interview in the interval between practice or an in-depth interview with key figures. The total number of interviews was 50.

### 3.4. Data Analysis and Representation

The first and second author transcribed the audio data from the interviews in full, leaving us with a number of transcripts that we read and re-read numerous times. In addition, the plentiful data written up as ethnographic field notes were also read in order familiarize ourselves with the main themes from the field. These were then directly compared with the interview transcripts, as these two sets of documents were coded around the core themes of: (1) Forms of space in the park culture and (2) the development of identity through Taijiquan PCS. These are represented in the subsequent sections, with the observational data being summarized in general descriptions of the setting and subculture, and the specific extracts of the interview transcripts representing the different voices of anonymized directors, instructors, key members, and ordinary members of distinct Taijiquan schools operating in the specific section of the Chengdu People’s Park. This approach differs from conventional ethnographic writing focusing on the field notes due to our emphasis on the first-person accounts from established (and often elderly) members of the Taijiquan communities. The work was translated from Mandarin into English and then checked over by a specialist English language consultant and a native British martial arts scholar and team member in a wider research project (the third author), who helped with the final representation of the article.

## 4. Production Process of Taijiquan Park Culture Space

### 4.1. Space Transformation: From “Public Space” in the Park to “Private Space” for the Exchange of Taijiquan Practice

As a kind of urban public space, urban parks are an organic part of the whole city. As Kohn pointed out, urban parks have the three characteristics of public property rights, equal access, and social interaction [28]. Different from the park’s general public space, the type of public space studied in this article, namely, Taijiquan PCS, refers to a specific physical area where the spontaneous organization of certain Taijiquan practice groups occurs and occupies the space for a long time, in addition to being a social area where people interact and communicate. In other words, Taijiquan PCS was a “special form” of public space in parks. Because of the Taijiquan practice, the “private space” was a result of converting “public space” in urban parks. Here, we describe the general transformation of Taijiquan PCS from the three dimensions of territorial consciousness, organizational consciousness, and assimilation of interaction.

#### 4.1.1. Territorial Consciousness

At its core, Taijiquan PCS is a physical space for practicing Taijiquan. Due to the limited public space in many parks, Taijiquan practitioners had to compete for a favorable position and then maintain a long-term occupancy; as a result of this, a “private space for Taijiquan practice” has formed. Their strong collective sense of “territory” was present [40]. From 6:30 to 8:30 in the morning and from 4:30 to 6:30 in the afternoon, several different groups of Taijiquan practitioners could be seen in the People’s Park. In an open area, there was a director and a speaker playing slow and melodious Taijiquan music. With slow music, the practitioners learned from the instructor’s direction and consistently practiced Taijiquan. Each Taijiquan group practiced at relatively fixed times and places in the park. In each group, they practiced different styles of Taijiquan in their own territory without interference. A group of Taijiquan practitioners came to the park a little late, and there were already several people shaking diabolos (a sport of Chinese minority nationalities) in the open field. The organizer (also a director) of the group came forward to negotiate and persuaded them to leave. The diabolos practitioners went on to practice in other areas without any objection. In several interviews following this observation, the research team asked the Yang style school about this agreement with other park culture groups over the specific training space:

*Q: This is a public place. Why did you turn them out? What did you tell them?*

*A: This site belongs to us. We have been practicing here for nearly ten years, and people who often come to the park are aware of it. Here is the Yang style Taijiquan training base. We all know each other, there is no need to argue. We are all friends but practice different aspects. (loud laugh) They are reasonable and understandable. (Leader Zhao, 24 February 2021)*

*Q: In this park, the three Taijiquan training groups all have a fixed area, time, and fixed members. How did you divide the training place? Is it naturally and consciously formed in this way?*

*A: Consciously? Impossible. As you know, the space in this park is limited and so many people exercise here. It’s not easy for our team to get such a good site (facing the lake with trees in back). Jokingly, by consultation or negotiation several times we have constantly “strived” for this fixed practice place. (Leader Zhao, 24 February 2021)*

*Q: Then why is it your area so big and comfortable? In this public place, why do the other Taijiquan practitioners agree?*

*A: Our group has the longest history and the largest number of members. Some members in the other two groups have transferred from our group. They are all aware of the rule. Even in public place, based on order of arrival, they should give way. Besides, we all exercise for joy and happiness. Nobody asks for trouble and wants to be unhappy! (Leader Zhao, 24 February 2021)*

*Q: What do you usually do if other people come and do exercise here?*

*Q: Let them go. We don’t care about other available times. But during our practice time, they should leave. We usually talk to them nice and peacefully. No conflict happens. It is not like square dance (big laugh). (Ordinary practitioner Liu, 1 March 2021)*

*Yes, it is true. We decide on our territory (loud laugh). (Ordinary practitioner Ma, 1 March 2021)*

Taijiquan PCS involved the redistribution of park space. It was a long-term struggle before it was fixed for a certain group. It was highlighted that instead of violence, they strived for it via “soft” struggle through consultation and negotiation. The territorial consciousness of Taijiquan PCS was the transformation process of the park’s public space on the basis of “rational discovery” [41], which is also the redistribution of an urban park’s public space.

The behaviors of the Taijiquan practice group were organized and purposeful. They were guided by Taijiquan practice habits and the direction of the practice’s purpose, objects, and symbols. They were motivated to form the Taijiquan PCS, which led to the transformation of public space into a special “private space.” Their territorial consciousness played a decisive role in the formation of Taijiquan PCS. To some degree, the transformation from the “public space” in the park to the “private space” for the exchange of Taijiquan practice was the domestication of the public space. Taijiquan PCS is not only a physical place for Taijiquan practice but also the result of the power struggle of territorial consciousness in public space.

#### 4.1.2. The Self-Organizational Consciousness

During the transformation of public space in the park, self-organizational consciousness and group action were the main expressive behaviors. Taijiquan practitioners were involved in the process of transforming “public space” in the park into a “private space” through Taijiquan practice, and they presented the characteristics of highly self-organized and unified activities. This study found that the three Taijiquan practice groups with different styles all had a high sense of self-organization and management and were generally grouped into different roles and identities. With this long-term self-management practice, a simple hierarchical structure of Taijiquan PCS was formed, which included instructors, key members, and ordinary practitioners, each with concrete responsibility descriptions. Generally, the instructor (leader) was an experienced Taijiquan master. He was also the manager or general director of Taijiquan PCS. Ordinarily, he arrived earlier and made all preparations for practice (including the sanitation of Taijiquan PCS, the adjustment of Taijiquan music, and the preparation of teaching and learning). In addition, he was also responsible for the audit and approval of new members. The key members had usually practiced Taijiquan longer than the others and had achieved a higher level of Taijiquan. They usually demonstrated and practiced in the front row. They performed the functions of managing and teaching when the director (the leader) was absent. As the main body of Taijiquan’s park space, ordinary practitioners were differentiated according to whether they were newcomers since Taijiquan practice has a certain threshold that requires a related foundation so that the main body is relatively stable and does not flow very often. The instructor (leader), along with key and experienced members, actively helped the newcomers learn and practice (reflect the characteristics of passing, helping, and leading). In this way, the newcomers obtained a sense of existence in Taijiquan PCS. The newcomers were the acquaintances or friends of the key or experienced members.

Linked with Taijiquan practice and built around the “Taijiquan master”, the self-organizational consciousness of Taijiquan PCS was a kind of self-management system that had a strong ability to self-organize. This self-management system can be labeled in terms of its strong organization and unity. Most Taijiquan practitioners arrived at the training area on time every day and carried out organized exercises under the unified guidance of the instructor (leader). We asked the research participants about these arrangements:

*Q: Every day, you do a lot of work for this group including hi-fi equipment preparations, spot arrangement, and the training content. You are also responsible for the teaching of the newcomers. Don’t you think it’s hard for you to do so much trivial work without any payment? Why are you willing to serve in this way?*

*A: Aha (loud laugh). It is not hard at all. I have just done what I am capable of. We have been in this team so long that we are all like in a big family. I feel more than delighted with their appreciation. Besides, it is easy to manage our team, as it is of a strong discipline. I am a retired old man with lots of spare time. Here I can practice, improve my Taijiquan skills, share the Taiji lifestyle, and also help the others. I am more than happy with it, not tired at all. (Leader Zhao, 24 February 2021)*

*Q: I have noticed that in this Taijiquan group, people all act in unity and have a strong sense of organization. How do you manage that?*

*A: Basically, we are retired. Some were teachers, some were civil servants, and some cadres. We have developed the strong sense of organization and ability from work. To run our Taijiquan practice team better, we (key members) have set up WeChat [Chinese messaging app] group, made “rules and regulations” and “membership fee system.” By comparing you will find out that the other two Taijiquan teams are not so well organized as we are. Isn’t it? (Leader Zhao, 24 February 2021)*

*Q: Could you please explain more about the “rules and regulations” and “membership fee system” you mentioned?*

*A: Actually, there are no formal or strict regulations, it is just some small rules, aiming to make everyone practice Taijiquan in a happy way here. For example, we should respect teachers, as they help us. What’s more, we should respect and help each other. Also, it is expected to abide by rules and actively participate in our activities. You could drop your request for leave in our WeChat group in case you are not available. In addition, we post the notices in WeChat group, you should check and reply in time. Everybody follows the rules. After all, we do it for our own good. (Key member Zhang, 24 February 2021)*

*As for the membership fee, we originally thought of paying for the teacher (such as leader and instructor) for his or her hard work, but the teacher refused. Instead of refunding, we use it for the sound maintenance or professional lecture delivered by a famous Taijiquan master. Our membership fee is very low, only 10 yuan a month, and it is not compulsory. Mr. Guo is in charge of the membership fee (he used to be an accountant in state-owned enterprises), and the accounts and expenses are clear (Key member Qu, 24 February 2021)*

High self-organization, unified management, and a clear structure regarding the team role allocations accelerated the transformation of Taijiquan PCS. It was also revealed that Taijiquan PCS was not only a “physical space” that was represented by the venue but also an “institutional space” that was linked with Taijiquan. Its transformation was multi-dimensional, including the “privatization” of a Taijiquan practice site or area and the institutionalization of self-organization and management of the Taijiquan group. The transformation process from the urban park’s public space into Taijiquan PCS can be treated as the process of isolation, invasion, and short-term replacement of urban public space.

#### 4.1.3. Assimilation of Interactive Topics

The assimilation of interaction in the Taijiquan group was one of the main characteristics in the process of transformation from a “public space” in the park to a “private Taijiquan space.” The group composition of Taijiquan PCS was complex. Driven by various motivations, the members also held different practice habits. Further, they were in different positions in Taijiquan PCS. However, undoubtedly during the formation process of Taijiquan PCS, a very different crowd gathered for Taijiquan practice, and gradually their interaction topics became similar or even equal. Once, after practicing a set of Taijiquan routines, the practitioners stopped to have a rest and started to actively converse. The conversation topics included how to improve the quality of Taijiquan movements, how to adjust breathing, where to buy and how to choose Taijiquan equipment and clothing, and Taijiquan competition performances. Sometimes they talked about something that had nothing to do with Taijiquan, such as their children’s work, their grandchildren’s school, as well as travel, purchasing real estate, and investment.

*Q: I realized that you usually had a rest when finished a set of Taijiquan routines, and then everyone was involved in a warm and active discussion. What were you mainly talking about? Is there any fixed or given topic?*

*A: We mainly talk about how to complete each Taijiquan movement with high quality. It is time for the newcomers to review and improve while we are resting. They could ask the experienced members for help on the essentials of each movement, the breathing style while practicing, and tips for choosing Taijiquan equipment. In a word, it’s all about Taijiquan practice. (Leader Zhao, 24 March 2021)*

*Q: But I noticed that you were also talking about something irrelevant to Taijiquan, such as your children’s work, grandchildren’s school, tourism, investment, and so on. These chats aren’t all about Taijiquan, are they?*

*A: Yes, yes, those you mentioned are experienced members. They have been practicing together for many years and shared a lot beyond Taijiquan. Hahaha (big laugh), we are not delivering a Taijiquan course, there is no need to be so rigorous, isn’t it? We are at about the same age and have similar life experience. Besides Taijiquan, we have a lot of common topics. (Leader Zhao, 24 March 2021)*

*Q: I found that you actively discussed how to practice Taijiquan during the rest but seldom participated in their chat (pointing to these experienced members). Aren’t you willing to chat with them or are you not interested in their topics?*

*A: I would like to, but my Taijiquan skill is poor. Instead of chatting with them, I have to ask for help and practice more during the break. I should not join their chat or be open to any competition until I have practiced well enough. (New practitioner Chen, 24 March 2021)*

*Sister Chen is too serious. She has practiced very well, although she just joined us one month ago. She is qualified for any competition or consultation. She should join us at the break. (Key member Zhang, 24 March 2021)*

*Q: Did you know each other before?*

*A: 1. No. We met here in this Taijiquan group, and now we became good friends basically. Ha ha (big laugh). (Experienced member Yang, 24 March 2021)*

*2. We used to only discuss Taijiquan, but now we talk about everything. (Ordinary member Hu, 24 March 2021)*

The “privatization process” of Taijiquan PCS was found to be a process of interaction between participants in the public space. The original and fixed interactive theme of Taijiquan was constantly broken or replaced by other themes. Although the themes of interaction presented diversity, it basically maintained a unified and common interactive discourse system. To some extent, this process of “assimilation of interactive topics” was a practical process of Taijiquan transforming “strangers” into “acquaintances” in Taijiquan PCS [42].

### 4.2. Space Expansion: “Physical Space” of Park Evolved to “Social Space” of Identity

The formation of Taijiquan PCS involved not only the transformation from the “public space” in the park into the “private space” of the Taijiquan practice but also the expansion from the “material space” of the Taijiquan practice into the “social space” of social interaction. The relationship between public space and Taijiquan was reflected in two aspects. First, the park’s public space was the material carrier of Taijiquan, which made the transformation from a “public space” to a “private space” feasible. Second, as a kind of space practice, Taijiquan transformed the material space into a social space, endowing it with social and cultural significance and making it an important field for the formation of urban culture and social relations.

Hannah Arendt put forward the following ideas: publicity is the embodiment of public space, the commonness of public space is the relevance of public life, the presence of public space, and the permanence of public space. The relevance of public life is worthy of being highlighted among them [43]. Taijiquan practice was the connection during the transformation from a “physical space” to a “social space”, which made Taijiquan PCS special. The Taijiquan practitioners voluntarily participated in shaping this special “public space”, which involved not only the process of constructing the subjectivity of Taijiquan PCS but also the process of constructing the identity of a Taijiquan practitioner. In Taijiquan PCS, the identity formation of “social space” mainly included the two dimensions of organizational identity and social identity.

#### 4.2.1. Organizational Identity: To Meet the Emotional Needs of Intimate Relationships through Taijiquan Practice

As was found in a previous study [44,45,46], the Taijiquan practitioners in the park in this study were mainly elderly. Most of them were born in the 1950s–1960s and shared similar life experiences: (1) they experienced the “Cultural Revolution” when in primary school or middle school, studied engineering and agriculture in middle school, and followed the movement of “educated youth supporting the countryside construction”; (2) when they were young during the era of the planned economy, most of them experienced the collective organization life; (3) they all witnessed China’s market-oriented reform and rapid economic development in the country. As time flies by, their collective life experiences inevitably still sit in their body and mind [47]. The collective consciousness and habits of collective expression pushed them to choose activities with the same attributes for social communication according to their experiences. In the process of Taijiquan group practice, the practitioners’ sense of collective belonging was satisfied, and their sense of organizational identity was also strengthened through Taijiquan PCS.

There are three Taijiquan training teams in the People’s Park. Distinguished by different Taijiquan schools, they are named team A, team B, and team C in order to protect their identities. The members were advised to voluntarily pay the membership fee of 20 yuan per month in team A. Team B and team C asked for no dues. However, team A was much larger than the other two teams and occupied a larger Taijiquan PCS. Through the interviews, we found out that Taijiquan practitioners were willing to pay dues, which made them become official members of the organization.

It was concluded that in this context, the “membership fee system” was essentially a kind of ceremony to join Taijiquan PCS, as well as a sign of entering the organization. With the help of these “dues”, individuals are recognized by the group organization and their sense of identification in the organization is greatly strengthened.

*Q: Does it make any difference if you don’t pay the membership fee?*

*A: Of course, paying the membership fee means you are a member of this team. In addition to Taijiquan activities, they also invite me to shop, dinner, tea gathering, and so on. I feel that my relationship with them is getting better and better. And in my eyes, the Taijiquan group is more like a big family. I feel very joyful and comfortable. Haha (big laugh)! (Ordinary practitioner Qing, 25 March 2021)*

*Yes, it makes great difference. The payment urges me to practice Taijiquan afterwards. In the past, I was so lazy that I missed the practice from time to time. Now it’s different. A certain members remind and encourage us to practice in the WeChat group. Now I am active with not only Taijiquan training, but also other activities organized. (Ordinary practitioner Huang, 25 March 2021)*

*Q: Without paying the membership fee, can’t the practitioner participate the other activities you mentioned?*

*A: Of course not, you are welcome in any activity. We are willing to pay, after all, it is not big sum of money. It’s just symbolic expenses. And everyone in our team is free to come and free to leave, there is no mandatory requirement. But we all enjoy ourselves being together. There’s no retreat.*

During the practice break, Taijiquan practitioners in the park talked about many topics, such as the treatment and prevention of disease, parenting and family relationships, fraud prevention skill sharing, and parenting tips regarding adjusting bad attitudes. Besides Taijiquan practice, many of them go shopping and dining, drink tea, mountain climb, and even travel together. In this way, they became good friends who talked about almost everything.

From the field observations, it was found that a new organization was formed through the link of Taijiquan. It not only met the basic communication needs of Taijiquan practitioners but also provided psychological support and material mutual assistance for the practitioners.

*Our organization is like a big family. No matter whoever meets difficulties, we shall help and support each other. For example, when Su’s wife was in hospital, each of us donated 100 yuan for help. Another example, when Zhao’s grandson went to school in Beijing, Ma asked his son in Beijing to welcome him. Such things happen quite often. (Leader Zhao, 28 March 2021)*

Linked by Taijiquan, the practitioners get to know each other. With the increasingly close communication between them, Taijiquan gradually built a mutual and intimate relationship of balance. This relationship enabled the Taijiquan practitioners to carry out emotional communication and to gain organizational recognition. While practicing Taijiquan, the “strangers” relationship gradually disappeared and a new “acquaintances” relationship developed in a new social platform bonding with Taijiquan. In other words, this is how the “physical space” of Taijiquan PCS developed into the “social space.” In addition, this is how each Taijiquan practitioner was accepted by the organization as a symbol of social communication.

#### 4.2.2. Social Identity: The Recognition of Others Promoted by Taijiquan in Social Relations

Taijiquan has been supported by the Chinese government as a way of promoting health since the founding of New China. In 1956, the State Sports Commission (now the General Administration of Sport of the People’s Republic of China) organized Taijiquan experts to identify the essence of Yang’s Taijiquan and formed the “Twenty-Four Style Taijiquan”, simplifying and standardizing the traditional Taijiquan [48]. In particular, the Outline of “Healthy China 2030” issued by the CPC Central Committee and the State Council on 25 October 2016 clearly points out that Taijiquan should be supported and promoted in extensive national fitness campaigns and a health project of Taijiquan should be implemented [49]. The legitimacy of Taijiquan fitness groups has strong social recognition in China. However, the practitioners of Taijiquan PCS pursued not only their emotional needs via intimate relationships and the organizational identity of group interactions but also continuously demanded the social identity of “the other” in society. This is the second dimension describing the expansion from a “physical space” to a “social space.” We named this the subjective construction through societal recognition of the others.

The Taijiquan practitioners had great enthusiasm for practicing Taijiquan itself, and they also had great enthusiasm and initiative for the social activities related to Taijiquan (Taijiquan competitions, community performances, community public health guidance, etc.). Competition, performance, and fitness guidance were no longer the simple individual pursuit of fitness but indicated their pursuit of social identity at a higher level. In their own words, “no matter [whether] you are the mentors, key members, or ordinary practitioners, everyone is given a stage to perform, and has the opportunity to show his or her value”. To many of the Taijiquan practitioners, participating in the social activities related to Taijiquan strengthened the cohesion of the organization and enabled them to gain a sense of joyful achievement. Each individual had the opportunity to construct a social identity and meet the universal appeal for the social identity of “the other”. The importance of this sense of performance in the construction of their specific Taijiquan identities is reflected in the words of people from all levels of the organisation, as seen in the following three extracts:

*I have never performed on stage during my life. It is amazing that I have won the prize in the Taijiquan competition at my age. I feel really proud of myself. It seems that practicing Taijiquan does not only keep me fit, but also satisfies my vanity. Aha (big laugh). (Ordinary practitioner Wu, 11 April 2021)*

*I quite admire Yang (the director and leader of this group). He practices Taijiquan so beautifully, and teaches so professionally. It is amazing that he always wins a prize in a competition. He is so popular and beloved. I wish I were him some day. LOL. (Key member Liu, 11 April 2021)*

*Haha (laugh), I am flattered. I can only play fairly well, they [the students] give me face, and look up to me. I shall make my best contribution whenever people need me. (Leader Zhao, 11 April 2021)*

Due to their daily practice driven by social recognition, Taijiquan practitioners were more likely to be motivated, to generate a sense of superiority, and to improve their Taijiquan skills. With the help of Taijiquan social activities (Taijiquan competitions, community performances, community public welfare fitness guidance, etc.), the social influence and recognition of the Taijiquan practitioners were further strengthened in the organization and in society. On the one hand, it enforced the group identity, enhanced the cohesion of Taijiquan PCS, and maintained its stable development. On the other hand, through constantly and actively constructing the identity of “the other”, which is widely recognized by society, the “social space” of Taijiquan PCS was expanded.

## 5. An Inner Mechanism of Self-Management in Taijiquan PCS

### 5.1. Communication and Consultation: The Democratic Operation Mechanism of Taijiquan PCS

The deliberative democratic mechanism works not only in the political discourse system but also in the discourse system of organizational governance. Jorge Valadez stated that communication and consultation is a kind of democratic governance form with great potential. It effectively responds to some core issues on intercultural dialogue and cognition within a multi-cultural society. It emphasizes the responsibility for the public interest and the promotion of mutual understanding in political discourse. In addition, it values and meets the need and interests of all people [50]. Taijiquan PCS realizes its self-management through free and equal dialogue, debate, negotiation, and deliberation. Among the participants in Taijiquan PCS, no matter whether they were leaders, core team members, or ordinary team members, everyone had equal opportunities for dialogue to realize the self-management on various issues in Taijiquan PCS. They reached agreements through democratic consultation on various issues, including the implementation of a “membership fee system”, the formulation and implementation of “rules and regulations”, and the task assignment in various organized social activities, such as competitions, performances, and community voluntary guidance. Through long-term development, Taijiquan PCS formed a sharing humanistic self-management model of democratic consultation. Although the director and key members are the main self-management team, everyone is reported to be equal to each other; together, they seek common ground that is good for everyone while reserving and tolerating differences. This is in contrast to a subordinate management mode with orders of superior and inferior.

### 5.2. Mutual Aid and Reciprocity: The Cooperative Operation Mechanism for the Autonomy of Taijiquan PCS

Taijiquan PCS formed a simple cultural space structure. Based on the fine distribution of different responsibilities and functions, the members were grouped into instructors, key members, and ordinary members. The different role positions determined that there must be a certain degree of disparity in their behaviorial logic. The director (leader) was the manager who was in charge of Taijiquan PCS. Generally, the key members demonstrated skills to the ordinary practitioners. In addition, in the absence of the mentor (leader), the key members took their place to manage and teach. Ordinary members were the main body of Taijiquan PCS. According to previous studies, the differences in role positioning and behavioral logic likely lead to the need for cooperation, as well as the possibility of conflicts between different subjects during the management of Taijiquan PCS [51]. However, under the principles of equality, mutual aid, and reciprocity, different members all cooperated regarding the decision making and implementation process of rules and organized various activities in Taijiquan PCS. In this way, the public’s interest in Taijiquan PCS was maximally realized. To put it in their way, “our Taijiquan training team is constructed by joint and cooperative management. Everyone is involved and helps. It is mutual assistance and reciprocity.”

### 5.3. Interests Balance: The Incentive Mechanism of the Autonomy in Taijiquan PCS

In the process of public space governance, various stakeholders have formed complex interest interactions and correlations. The key to the self-management of Taijiquan PCS lay in the formation of interest exchange and balance mechanisms among all interested subjects. It integrated the resources of all parties, meeting the interest demands of all subjects at the same time, and realized the needs of the development of Taijiquan PCS. The instructor had a relatively high social status in Taijiquan PCS with a high reputation, authority, and discursive right. It was through organizing and guiding of Taijiquan practitioners that the instructor’s social identity was realized and personal satisfaction was obtained. By serving others, the key members gained recognition from others, which also helped to construct their value of existence in Taijiquan PCS and obtained to obtain a personal sense of achievement and satisfaction. Through participating in activities in Taijiquan PCS, ordinary members improved their own welfare, grew a sense of belonging, and obtained group and social identities.

## 6. Discussion

There is an abundance of experimental research on Taijiquan in indoor laboratories that have investigated the physical and mental health benefits of the art. However, in many parts of the world, the word “Taijiquan” is often associated with images of elderly Chinese people moving slowly, en masse, in urban parks, which is increasingly the case in mainland China. Yet, to date, there has been little research on the meanings behind real-life group practice in city parks in China and other countries. This article was the first output from an ethnographic study that sought to share the voices of different teachers, members and organizers of various Taijiquan groups in Chengdu, China. Mass Taijiquan practice was selected due to the prominence of the different groups in a desirable location in the park which has been negotiated thanks to the long history of certain groups in this public place.

In terms of theory, Lefebvre was one of the first scholars to systematically expound on the concept of space. The most important aspect of his space concept is the reflection and criticism of capitalist social space. His proposed spatial practice and representations of space provide a new starting point for urban research. This conceptualization emphasizes the significance of space practice in communicating the relationship between the city and people and points out that urban social life is unfolding in the urban space. Spatial metaphors, such as location, status, position, region, field, and boundary, all reveal the social boundaries and resistance, as well as the boundary between the subject’s construction of self and alien and provide the division of urban classes from the “spatial dimension” to form relevant subjects [25]. In a sense, Taijiquan PCS constructed in this article was also a kind of urban space. Taijiquan PCS can be interpreted using Lefebvre’s “space ternary dialectics”.

The “spatial practice” of Taijiquan PCS refers to the production of space, which not only involved the various material practice activities and behaviors of Taijiquan practitioners but also the results of Taijiquan activities and behaviors. This kind of space practice was a concrete and empirical space, a kind of “perceived space”, which included the conversion of the “public space” of the city park to the “private space” of Taijiquan practice. The “material space” of the city park was an increase in the dimension of the “social space” of social identity.

The “representations of space” in Taijiquan PCS defined a conceptual space, a space conceived by our researchers, and an abstract space as our research object. Although the “space appearance” of Taijiquan PCS seldom reflected the practical influence of the ruling group intervening and changing the spatial structure, the operating mechanism and autonomous mechanism of Taijiquan PCS made the Taijiquan practice an “orderly” social activity. In a sense, Taijiquan PCS was also a space for the governance of Taijiquan groups. As a result, Taijiquan PCS became, as Lefebvre said, a tool of rule to protect the interests of the ruling class and this conceived space became a kind of implicit spatial power.

Space is a social product. On the one hand, Taijiquan practitioners interacted in Taijiquan PCS to realize the transformation and reorganization of Taijiquan PCS. Taijiquan PCS was a medium for interaction between Taijiquan practitioners. On the other hand, the interactive relationship that formed between Taijiquan practitioners promoted the change of spatial attributes, further promoted the reproduction of Taijiquan PCS, and demonstrated the inter-construction characteristics of the Taijiquan practitioners and Taijiquan PCS.

Viewing Taijiquan PCS from the perspective of space production, the relationship between Taijiquan PCS and Taijiquan was embodied at three levels. First, Taijiquan PCS was the material carrier of Taijiquan. Second, as a kind of physical practice in Taijiquan PCS, Taijiquan transformed the “public space” of the park into the “private space” of the Taiji practitioners and then became an important fitness field for Taiji practitioners. Third, as a social practice in Taijiquan PCS, Taijiquan transformed the physical space into a social space, endowed the space with social and cultural significance, and made it a social interaction field of new urban social relations.

## 7. Conclusions

The production of Taijiquan PCS was not only an upgrade from material space to a social and cultural space but also a social activity that was spontaneously organized through the link of Taijiquan practice. Through Taijiquan park activities, a series of new social relations, interpersonal relations, and human–Earth relations were generated. The new relationship network constructed in Taijiquan PCS was not a top-down hierarchy but was a balanced relationship between the Taijiquan practitioners and society. It was presented in terms of the following three aspects.

First, it met the physical health needs of the Taijiquan practitioners through participating in the production activities of Taijiquan PCS. Logically, the pursuit of physical fitness was the start of the production of Taijiquan PCS. A healthy life was one of the main outcomes expressed by the group in Taijiquan PCS. The practitioners paid attention to not only fitness methods but also a healthy diet because of participating in activities in Taijiquan PCS. Further, the practitioners replaced their previous entertainment methods, such as watching TV and playing Majiang (Majong), with Taijiquan practice. Through interviews, it was found that in the urban areas, the elderly with a certain material basis were paying more attention to their welfare and leisure. Taijiquan PCS became an exercise space for their welfare and keeping fit.

Second, the practitioners’ senses of collectivism and desire for belonging were satisfied by participating in the production activities of Taijiquan PCS. Taijiquan PCS offered a pleasant place for their communication. The vast majority of participants in Taijiquan PCS were middle-aged people and elderly retirees. The latter age group’s social relations are constantly weakened as they “exit” from the familiar work environment, and the biggest problem they are facing is spiritual loneliness and the sharp decline of social contacts [52]. What was more important than fitness was that through Taijiquan PCS, the practitioners made friends, explored a social space for communication, regained a sense of collective belonging, and satisfied their emotional needs. It is believed that Taijiquan PCS reshaped the interpersonal relationship paradigm of China’s middle-aged and elderly society.

Third, Taijiquan practitioners built their identity of “the other” by participating in the production activities of Taijiquan PCS, which was a place for them to pursue different demands of identification. Although for most Taijiquan practitioners, the “lack” of social communication after retirement meant they risked being short of places for social relations, Taijiquan PCS provided them with places for social interaction and enabled them to develop a strong group and social identity. No matter whether they were the leader, a key member, or an ordinary member, they all pursued the identity of “the other” in Taijiquan PCS. Therefore, the production process of Taijiquan PCS was the construction process of Taijiquan practitioners’ identity.

China is a society, like many others, that values human connection and relation. The rapid modernization process of reform and opening up not only accelerated China’s urbanization but also speeds up the demise of the interpersonal relationship of the “acquaintance society” in agricultural society. In fact, after the reform and opening up, with the decline of collectivism and the rise of modernism, rational and orderly urban planning replaced the intimate relationships found in the acquaintance society, and a culture of strangers became the mainstream for social relations [53]. The production of Taijiquan PCS was a process of “re-acquaintance” of micro-social relations in cities of China.

Through bonding with Taijiquan practice, Taijiquan PCS involves the reproduction of urban public space. It was not only Taijiquan practitioners’ reconstruction and derivation of urban public space but also a historical memory of Taijiquan practitioner groups and the return of the collective life experience. Furthermore, it was the construction of Taijiquan practitioner groups’ identification and the development of self-organization.

The high degree of autonomy of Taijiquan PCS provides a guarantee for its stable and sustainable development. The democratic autonomy of communication and consultation, the cooperative autonomy of mutual assistance and reciprocity, and the incentive autonomy of balanced interests were the internal mechanisms of Taijiquan PCS. The autonomous mechanisms of Taijiquan PCS made it “orderly” in the urban public space.

## 8. Limitation

Through ethnographic research on Taijiquan practitioners in one public park in Chengdu, China, this article discusses the production process, autonomous mechanisms, and identification of participants in Taijiquan PCS. However, due to the lack of in-depth detailed research with groups of the Taijiquan practitioners regarding ages, genders, educational backgrounds, and life experiences, this study did not investigate the practical space of different groups of Taijiquan PCS. In follow-up research, it is necessary to refine the different groups of Taijiquan practitioners to present the diversity of spatial practice of specific Taijiquan practicing groups in a more in-depth way and obtain a deeper understanding of the characteristics of different types of Taijiquan practitioners, such as subjectivity, initiative, and self-organization. In addition to this, we hope to speak with the other groups in the park in terms of how they perceive the Taijiquan PCS and their relationship to this. As such, this article is the first output of an emerging study examining park culture, space, and the place of Taijiquan within them.

## Data Availability

The data presented in this study are available on request from the corresponding author. The data are not publicly available due to the ethical agreement with the Chengdu Sport University Social Sciences Ethics Panel to keep the data under Ma Xiujie’s personal OneDrive account, which is not accessible to the wider public.
